# Evaluating the Risk of Paroxysmal Atrial Fibrillation in Noncardioembolic Ischemic Stroke Using Artificial Intelligence-Enabled ECG Algorithm

**DOI:** 10.3389/fcvm.2022.865852

**Published:** 2022-04-08

**Authors:** Changho Han, Oyeon Kwon, Mineok Chang, Sunghoon Joo, Yeha Lee, Jin Soo Lee, Ji Man Hong, Seong-Joon Lee, Dukyong Yoon

**Affiliations:** ^1^Department of Biomedical Systems Informatics, Yonsei University College of Medicine, Yongin, South Korea; ^2^VUNO Inc., Seoul, South Korea; ^3^Department of Neurology, Ajou University School of Medicine, Suwon, South Korea; ^4^Center for Digital Health, Yongin Severance Hospital, Yonsei University Health System, Yongin, South Korea; ^5^BUD.on Inc., Seoul, South Korea

**Keywords:** atrial fibrillation, noncardioembolic ischemic stroke, artificial intelligence, electrocardiogram, deep neural network, regression analysis

## Abstract

**Background:**

The identification of latent atrial fibrillation (AF) in patients with ischemic stroke (IS) attributed to noncardioembolic etiology may have therapeutic implications. An artificial intelligence (AI) model identifying the electrocardiographic signature of AF present during normal sinus rhythm (NSR; AI-ECG-AF) can identify individuals with a high likelihood of paroxysmal AF (PAF) with NSR electrocardiogram (ECG).

**Objectives:**

Using AI-ECG-AF, we aimed to compare the PAF risk between noncardioembolic IS subgroups and general patients of a university hospital after controlling for confounders. Further, we sought to compare the risk of PAF among noncardioembolic IS subgroups.

**Methods:**

After training AI-ECG-AF with ECG data of university hospital patients, model inference outputs were obtained for the control group (i.e., general patient population) and NSRs of noncardioembolic IS patients. We conducted multiple linear regression (MLiR) and multiple logistic regression (MLoR) analyses with inference outputs (for MLiR) or their binary form (set at threshold = 0.5 for MLoR) used as dependent variables and patient subgroups and potential confounders (age and sex) set as independent variables.

**Results:**

The number of NSRs inferenced for the control group, cryptogenic, large artery atherosclerosis (LAA), and small artery occlusion (SAO) strokes were 133,340, 133, 276, and 290, respectively. The regression analyses indicated that patients with noncardioembolic IS had a higher PAF risk based on AI-ECG-AF relative to the control group, after controlling for confounders with the “cryptogenic” subgroup having the highest risk (odds ratio [OR] = 1.974, 95% confidence interval [CI]: 1.371–2.863) followed by the “LAA” (OR = 1.592, 95% CI: 1.238–2.056) and “SAO” subgroups (OR = 1.400, 95% CI: 1.101–1.782). Subsequent regression analyses failed to illustrate the differences in PAF risk based on AI-ECG-AF among noncardioembolic IS subgroups.

**Conclusion:**

Using AI-ECG-AF, we found that noncardioembolic IS patients had a higher PAF risk relative to the general patient population. The results from our study imply the need for more vigorous cardiac monitoring in noncardioembolic IS patients. AI-ECG-AF can be a cost-effective screening tool to identify high-risk noncardioembolic IS patients of PAF on-the-spot to be candidates for receiving additional prolonged cardiac monitoring. Our study highlights the potential of AI in clinical practice.

## Introduction

Atrial fibrillation (AF) is a well-known risk factor for ischemic stroke (IS) and is associated with a five-fold increase in stroke incidence (1–3). IS patients with identified AF without relevant arterial stenosis or suspected lacunar syndrome can be classified as cardioembolic in etiology (4). However, given the often asymptomatic nature of AF (5, 6), it is sometimes unrecognized at the time of stroke (7, 8). Misclassification of stroke etiology because of failure in detecting AF may be devastating for the patient, as it may lead to recurrent strokes (1, 9, 10). AF detection may be especially important in cryptogenic stroke patients for whom imaging results suggest an embolic etiology, although no potential source is identified. However, short- to medium-term cardiac monitoring might not be long enough to sufficiently reveal previously undetected AF, while long-term cardiac monitoring with insertable cardiac monitors (ICM) is limited by high cost and the invasiveness of the implant procedure (11). Furthermore, noncardioembolic stroke with determined etiologies may increase the rate of undetected AF, as non-atrial stroke mechanisms and AF may share common vascular risk factors (12). Therefore, such concerns raise the question about the target population for whom ICM should be considered. An alternative method to non-invasively assess AF risk among patients with noncardioembolic IS on-the-spot would be of great clinical significance.

A recent study demonstrated that an artificial intelligence (AI) model could identify individuals with a high likelihood of paroxysmal AF (PAF) using their normal sinus rhythm (NSR) electrocardiogram (ECG) (13). This simple, non-invasive, and inexpensive test could have significant clinical implications for AF screening as it has the potential to identify patients with high AF risk, thereby enabling additional prolonged cardiac monitoring. An elevated risk of PAF in patients with noncardioembolic IS, if observed using the aforementioned AI model identifying the electrocardiographic signature of AF present during NSR (AI-ECG-AF), could affect post-stroke ECG monitoring strategies among such patients and also demonstrate the importance of AI algorithms in disease screening, prediction, and management.

Thus, in this study, we aimed to retrospectively compare the risk of PAF between noncardioembolic IS patients and general patients of Ajou University Medical Center (AUMC) using AI-ECG-AF. Further, we sought to compare the risk of PAF among noncardioembolic IS subgroups.

## Methods

### Data Sources and Study Population

Noncardioembolic IS patients were identified from AUMC's institutional stroke registry, which prospectively collects relevant data, including stroke etiologies of all hospitalized stroke patients. For all stroke patients, 48-h ECG monitoring in the stroke unit, 24-h Holter monitoring, and transthoracic echocardiography were routinely performed to screen for cardiac sources of embolism. From this database, we identified noncardioembolic IS patients admitted between March 2008 and August 2018. Specifically, IS attributed to large artery atherosclerosis (LAA), and small artery occlusion (SAO), or cryptogenic (“cryptogenic embolism” or “other cryptogenic”) IS according to the Stop Stroke Study Trial of Org 10172 in Acute Stroke Treatment (SSS-TOAST) classification were included (4, 14). In these patients, corresponding ECG data were sourced from the AUMC's institutional ECG database. Patients' data were included in the final analysis only when their NSR ECG was available.

**Figure 1:**
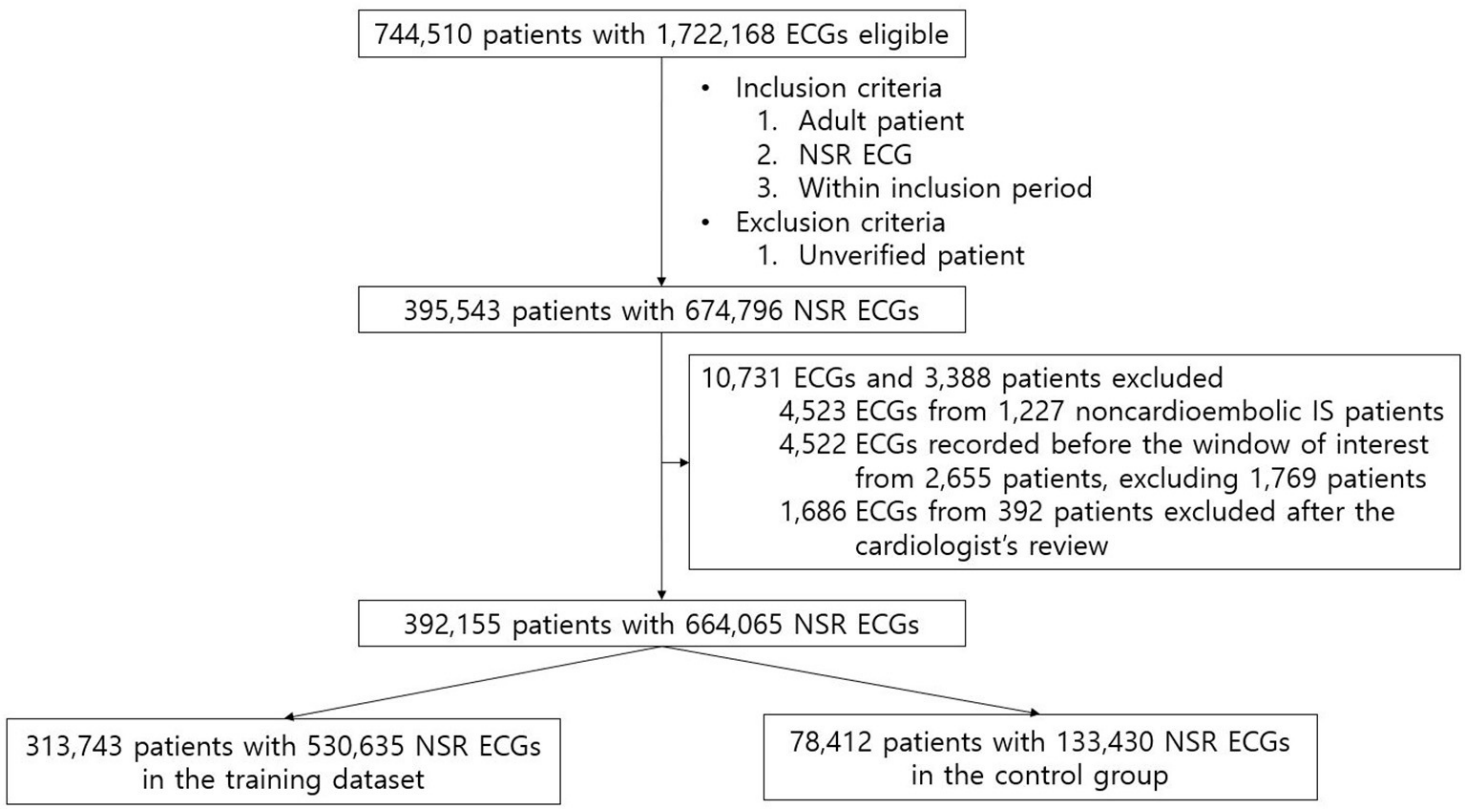
Flow diagram of the patients' data included in the study. After applying all the exclusion criteria, 392,155 patients with 664,065 NSR ECGs remained, which were then randomly split into the training dataset and the control group with a ratio of 8:2.

For AI-ECG-AF training and validation, we utilized standard 12-lead ECG data from AUMC's institutional ECG database originally extracted from the General Electric Healthcare MUSE^TM^ system (Figure 1; Supplementary Methods). The length of each ECG was 10 s. For our study, we used all the ECGs acquired within the inclusion period (between June 1994 and August 2018) from all the adult (age ≥18) patients who measured the standard 12-lead ECG at AUMC. We referred to the automatic interpretations provided by the ECG machine for extracting NSR and AF ECGs. NSR ECGs were defined as cases where automatic interpretations included “normal sinus rhythm,” “sinus bradycardia,” or “sinus tachycardia,” while containing no phrases related to any abnormality. AF ECGs were defined as cases where the automatic interpretations included “atrial fibrillation” or “atrial flutter,” while not containing the following phrases: “lead reversal,” indicating that the leads might have been misplaced; “poor quality,” implying that the ECG contains artifacts; “pacemaker,” signifying that an artificial pacemaker might be present. We employed the following methods to identify and label study groups. To reduce ambiguity, patients with a diagnosis code for AF in the electronic medical records (EMR) database of AUMC but no AF ECG record were considered to have unverified AF and were excluded. NSR ECGs with at least one AF ECG and no AF ECG recorded from the same patient were classified as positive and negative for AF, respectively. NSR ECGs classified as positive for AF that were measured 31 days before the first recorded AF (i.e., measured before the window of interest) were excluded on the assumption that although structural changes associated with AF would be present before the first recorded AF, these structural changes would not have developed at a distant time in the past since the first recorded AF. Next, to improve the accuracy of data labeling, a cardiologist (MC) reviewed the raw waveforms of all the AF ECGs of patients classified as positive for AF and excluded patients with raw waveforms of the AF ECGs not considered to be actually AF. The NSR ECGs of patients with noncardioembolic IS were also excluded. The remaining NSR ECGs after applying all the aforementioned inclusion and exclusion criteria were split into the AI-ECG-AF training dataset and an independent hold-out dataset that was considered to represent AUMC's general patient population and used as the control group in the regression analyses, with an 8:2 ratio.

### AI Model Development and Performance Evaluation

Only the raw waveforms of the standard 12-lead ECG were used as the input for the model. We constructed a convolutional neural network based on residual networks (15). The architecture of our model is depicted in Supplementary Figure 1. The details of data preprocessing and the neural network architecture are described in Supplementary Methods.

We randomly divided the training dataset into five-folds and performed five-fold cross-validation to select the best hyperparameters. The models with the best validation performances were chosen in each fold of the five-fold cross-validation. During model inference (on the control group and the NSR ECGs from patients with noncardioembolic IS), output probability was obtained for each of the five chosen models and the average probability was used as the final inference output.

A receiver operating characteristics (ROC) curve of the AI-ECG-AF was created for the control group and the area under the ROC curve was calculated. We computed the accuracy, sensitivity, specificity, positive and negative predictive values, and F1 score of the AI-ECG-AF when the threshold was set at 0.5 by convention. The aforementioned performance metrics were calculated not only for the first recorded NSR ECG per patient in the control group to mimic a real screening scenario but also for all the NSR ECGs in the control group.

### Comparison of AF Risk Between Groups

After the AI-ECG-AF development, model inference outputs were obtained for the NSR ECGs of the control group and those that were measured within seven days before or after the admission date of noncardioembolic IS patients. The inference outputs could be considered to represent the relative probability of PAF presence in each NSR. The time window of seven days before or after the admission date of noncardioembolic IS patients was selected to accurately reflect the state of the patient at the time of stroke occurrence.

We conducted MLiR and MLoR analyses with the AI-ECG-AF's inference outputs (for MLiR) or their binary form (for MLoR, the threshold set at 0.5) set as the dependent variables and patient subgroups (LAA, SAO, cryptogenic or control group, with the control group set as the reference) and potential confounders (age and sex) set as independent variables. First, to compare PAF risk based on AI-ECG-AF between noncardioembolic IS patients and the control group, noncardioembolic IS patients' data and the control group data were included in the regression analyses. Second, to compare PAF risk based on AI-ECG-AF among noncardioembolic IS subgroups, data for only patients with noncardioembolic IS were included in the analyses. The methods and results for checking the overall significance or goodness of fit of these regression models are specified in Supplementary Methods. Statistical comparisons of dataset characteristic distributions are specified in the Supplementary Methods, Supplementary Table 1, and Supplementary Figures 2–7. All statistical analyses were performed with the R Statistical Software (version 4.0.4; R Foundation for Statistical Computing, Vienna, Austria). *P*-value < 0.05 was considered significant in all tests.

## Results

### Dataset Characteristics

**Figure 2:**
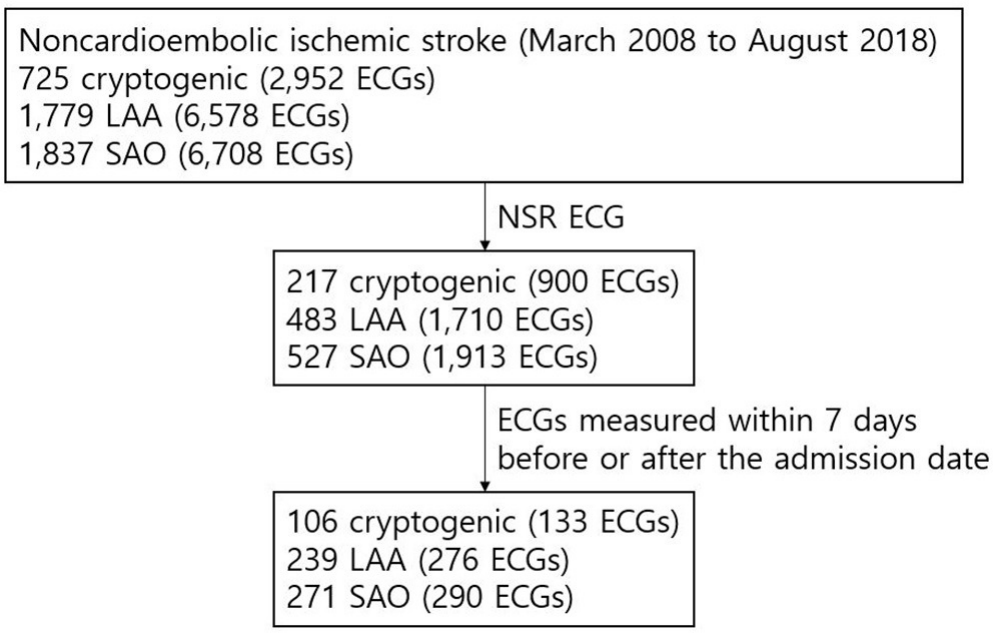
Flow diagram of the ECGs of patients with noncardioembolic IS. The NSR ECG data within 7 days before or after the admission date was available from the institutional stroke registry for 106, 239, and 271 patients classified as “cryptogenic,” “LAA,” and “SAO” strokes, respectively, and were included in the final analysis.

Table 1 presents the characteristics of the training dataset and of the patients from whom AI-ECG-AF's inference outputs were obtained. The training dataset had 313,743 patients (44.7% males and 55.3% females) with 530,635 ECGs (mean age 48.01 ± 14.75). The control group comprised 78,412 patients (44.6% males and 55.4% females) with 133,430 ECGs (mean age 48.01 ± 14.72). The share of ECGs positive for AF in both the training dataset and the control group was 0.9%. Supplementary Table 2 shows the number of ECGs ordered in each medical department in the control group. Figure 2 illustrates the flow diagram of the ECGs from patients with noncardioembolic IS. A total of 725, 1,779, and 1,837 patients from AUMC's stroke registry from March 2008 to August 2018 were classified as “cryptogenic,” “LAA,” and “SAO” strokes, respectively. Out of these patients, 217 “cryptogenic,” 483 “LAA,” and 527 “SAO” patients had at least one NSR ECG recorded within the inclusion period (June 1994 to August 2018). The number of NSR ECGs measured within seven days before or after the admission date of these patients were 133, 276, and 290 from 106 “cryptogenic,” 239 “LAA,” and 271 “SAO” patients, respectively.

**Table 1:** Dataset characteristics.

	**Training dataset (*n =* 530,635)**	**Control** **(*n =* 133,430)**	**Cryptogenic (*n =* 133)**	**LAA (*n =* 276)**	**SAO (*n =* 290)**	***p*-value**
Number of patients	313,743	78,412	106	239	271	
Sex
Males (%)	140,312 (44.7)	34,982 (44.6)	62 (58.5)	157 (65.7)	159 (58.7)	<0.001
Females (%)	173,431 (55.3)	43,430 (55.4)	44 (41.5)	82 (34.3)	112 (41.3)	
Age	48.01 ± 14.75	48.01 ± 14.72	62.65 ± 13.93	66.79 ± 12.33	64.29 ± 11.27	<0.001
Positive for AF (%)	4,723 (0.9)	1,169 (0.9)				

*Each noncardioembolic IS subgroup had a significantly higher proportion of males and higher mean age than the control group*.

### AI Model Performances

**Figure 3:**
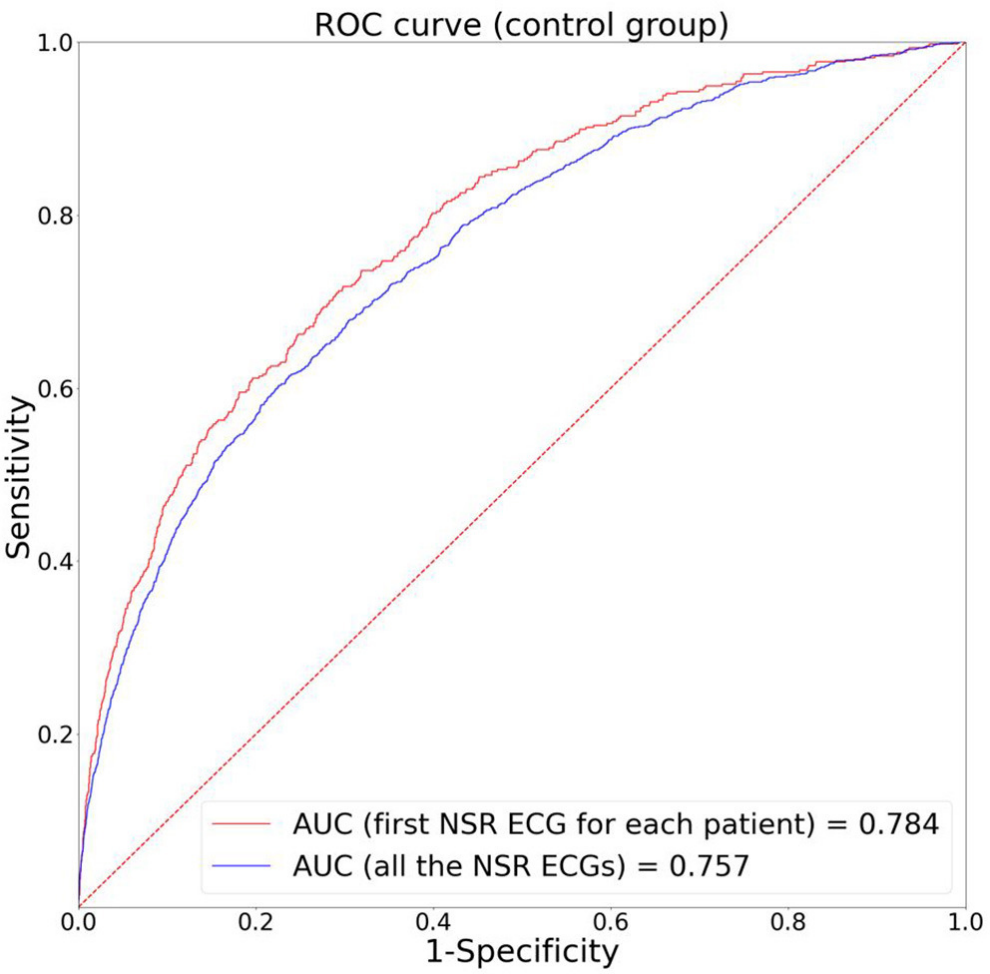
Performance of the AI model.

Figure 3 depicts the ROC curves of AI-ECG-AF for the control group. The area under the ROC curve for the first recorded NSR ECG per patient was 0.784 and that for all the NSR ECGs was 0.757. Supplementary Table 3 presents the accuracy, sensitivity, specificity, positive and negative predictive values, and F1 score of AI-ECG-AF when the threshold was set at 0.5.

### Regression Analyses Results

Table 2 illustrates the results of the regression analyses when the control group and noncardioembolic IS patients' data were included in the regression analyses. All the independent variables (age, male sex, cryptogenic, LAA, and SAO) were statistically significantly associated with the dependent variables, with positive β-coefficient values for all the SSS-TOAST subgroups, indicating that noncardioembolic IS patients had a higher PAF risk based on AI-ECG-AF compared to the control group after controlling for confounders. For the subgroups, the “cryptogenic” subgroup had the highest β-coefficient value, followed by “LAA” and “SAO” subgroups. Specifically, the wwodds ratio (OR) of inference output ≥0.5 compared to the control group was 1.974 (95% confidence interval [CI]: 1.371–2.863) for the “cryptogenic” subgroup, 1.592 (95% CI: 1.238–2.056) for the “LAA” subgroup, and 1.400 (95% CI: 1.101–1.782) for the “SAO” subgroup. The MLoR results remained nearly consistent at different thresholds for the inference output (Supplementary Table 4). All the independent variables were statistically significant at a significance level of 0.05 at thresholds of 0.35, 0.40, 0.45, and 0.55 for the inference output; except for the “SAO” subgroup, which was marginally significant (i.e., *p*-value between 0.05 and 0.1), all other independent variables were statistically significant at a significance level of 0.05 at the threshold of 0.60 for the inference output; for the patient subgroups, “cryptogenic” subgroup had the highest β-coefficient values at all the thresholds.

**Table 2:** Regression analysis results when the control group and noncardioembolic IS patients' data were included in the analyses.

**Variable**	**β**	**SE**	**95% CI of β**	**OR**	**95% CI of OR**	***p*-value**
**Multiple linear regression results**
Age	0.00545	0.0000324	0.00538–0.00551			<0.001
Sex
Female	Reference					
Male	0.0243	0.000956	0.0224–0.0262			<0.001
Patient subgroup
Control	Reference					
Cryptogenic	0.0744	0.0151	0.0447–0.104			<0.001
LAA	0.0413	0.0105	0.0207–0.0620			<0.001
SAO	0.0344	0.0103	0.0143–0.0545			<0.001
**Multiple logistic regression results**
Age				1.053	1.052–1.054	<0.001
Sex						
Female				Reference		
Male				1.280	1.249–1.313	<0.001
Patient subgroup
Control				Reference		
Cryptogenic				1.974	1.371–2.863	<0.001
LAA				1.592	1.238–2.056	<0.001
SAO				1.400	1.101–1.782	0.006

*Compared to the control group, all stroke etiology subgroups exhibited a significantly higher PAF risk based on AI-ECG-AF*.

Table 3 presents the results when only noncardioembolic IS patients' data were included in the regression analyses. Compared to the SAO subgroup, the β-coefficients for the LAA subgroup were not statistically significant at a significance level of 0.05 for both regression models, and those for the cryptogenic subgroup were statistically significant only for MLiR. At various thresholds for the inference output in MLoR, the β-coefficients for both the LAA and cryptogenic subgroups were not statistically significant, failing to display differences in PAF risk based on AI-ECG-AF among noncardioembolic IS subgroups (Supplementary Table 5). Model inference outputs obtained for the NSR ECGs of the control group and noncardioembolic IS patients are provided in Supplementary Table 6.

**Table 3:** Regression results when only noncardioembolic IS patients' data were included in the analyses.

**Variable**	**β**	**SE**	**95% CI of β**	**OR**	**95% CI of OR**	***p*-value**
**Multiple linear regression results**
Age	0.00496	0.000555	0.00386–0.00605			<0.001
Sex
Female	Reference					
Male	0.0378	0.0140	0.0104–0.0653			0.007
IS subgroup						
SAO	Reference					
Cryptogenic	0.0391	0.0186	0.00260–0.0757			0.036
LAA	0.00731	0.0150	−0.0222–0.0368			0.627
**Multiple logistic regression results**
Age				1.049	1.034–1.063	<0.001
Sex
Female				Reference		
Male				1.234	0.890–1.712	0.208
IS subgroup
SAO				Reference		
Cryptogenic				1.395	0.904–2.168	0.135
LAA				1.151	0.812–1.632	0.428

*The threshold for the inference output (dependent variable) was set at 0.5 for the multiple logistic regression. The inference output of 80 (60.2%), 168 (60.9%), and 158 (54.5%) cases was ≥0.5 for cryptogenic, LAA, and SAO subgroups, respectively*.

## Discussion

In this study, we trained and validated AI-ECG-AF and used it to compare the risk of PAF between noncardioembolic IS patients and general patients. The regression analyses demonstrated that based on AI-ECG-AF, noncardioembolic IS patients had a higher PAF risk relative to the control group, after controlling for confounders: For the SSS-TOAST subgroups, the “cryptogenic” subgroup had the highest OR followed by the “LAA” and “SAO” subgroups. However, subsequent regression analyses failed to illustrate the differences in PAF risk based on AI-ECG-AF among noncardioembolic IS subgroups.

Using AI-ECG-AF, the increased PAF risk in noncardioembolic IS demonstrated in our study supports the recent shifts in IS guidelines that encourage vigorous cardiac monitoring. Patients with embolic stroke of undetermined source (ESUS) carry a substantial annual stroke recurrence rate of 3–6% despite antithrombotic therapy (16, 17). However, the empirical use of oral anticoagulants for secondary prevention of stroke without identification of AF has not proven to be superior to aspirin and increases the risk of bleeding (18, 19), thus rendering the identification of AF important for guiding therapy. Accordingly, a recent guideline by the European Society of Cardiology recommends short-term ECG recording followed by continuous ECG monitoring for at least 72 h (Class I recommendation), and additional ECG monitoring through long-term non-invasive ECG monitors or ICM (Class IIa recommendation) (20). A guideline in the United States recommends cardiac monitoring for at least 24 h in patients with IS (Class I recommendation) (21), and another recommends an ICM in patients with cryptogenic stroke when external ambulatory monitoring is inconclusive (Class IIa recommendation) (9).

Our study results did not indicate a difference in risk among noncardioembolic IS subgroups, which is aligned with the findings in another recent study that compared PAF risk between the embolic stroke of undetermined source and other mechanisms of stroke (excluding AF) using AI-ECG-AF and illustrated no association (22). Although the relatively small number of patients with NSR ECGs may have limited our statistical significance, a recent clinical trial reported AF detection rates of up to 12.1% with ICM during a period of 12 months in patients with IS attributed to LAA or SAO (23). Even patients with IS classified as noncardioembolic in etiology may have an increased risk of subclinical AF (7, 8, 23). This is reasonable as non-atrial stroke mechanisms and AF may share common vascular risk factors (12). While the clinical significance of this finding calls for further research, such results raise the question about the target population for whom ICM should be contemplated.

While AI-ECG-AF cannot be considered a confirmatory test because its accuracy is not yet close to 100%, it is an efficient screening tool that can non-invasively assess PAF risk on-the-spot using only ECG as input. Previous studies have been conducted, or are being conducted, using AI-ECG-AF or a similar AI model. Raghunath et al. (24), have trained an AI model predicting new-onset of AF from the 12-lead ECG and have demonstrated that the model may help identify patients at risk of AF-related strokes. Further, an ongoing clinical trial is aiming to prospectively validate an AI-ECG-AF by enrolling patients with AI-ECG-AF predicted risk of PAF and providing them with continuous cardiac monitoring devices to observe AF burden (25).

If AI-ECG-AF is well validated, it can be a cost-effective screening tool to identify high-risk noncardioembolic IS patients of PAF on-the-spot as candidates for receiving additional prolonged cardiac monitoring. For AF identification, short- to medium-term cardiac monitoring might not be long enough to sufficiently reveal previously undetected AF (7, 8, 23), while long-term cardiac monitoring is expensive and invasive (26). Moreover, considering the low yield of AF in clinical trials targeting ESUS patients, the cost-effectiveness of long-term cardiac monitoring may be questionable, and whether more widespread adoption of ICMs translates into health economic benefits remains unproven (27). The cost-effectiveness of long-term cardiac monitoring could be improved if low-risk patients could be excluded (28). With AI-ECG-AF, high-risk noncardioembolic IS patients of PAF can be selected to receive additional cardiac monitoring. The threshold of the AI-ECG-AF's inference outputs for the selection of patients for additional prolonged cardiac monitoring at which the health economic benefits are maximized would need further prospective studies to be determined. Our study, although retrospective, showed that AF risk was higher in noncardioembolic IS patients and can serve as a basis for such prospective validation.

Our study has some limitations. First, we did not add potential confounders other than age and sex to the regression analyses. Including more confounders that can be risk factors for PAF and possibly have varying distributions across different patient subgroups, such as those with hypertension or diabetes mellitus, would be ideal. However, incompleteness is a frequently occurring problem in EMRs (29). For example, the absence of any diagnostic code in the EMR does not preclude the absence of the disease because the patients' thorough evaluation and all the relevant information entered into the EMR cannot be guaranteed. Thus, we excluded such data from the analysis. Second, the performance of the AI-ECG-AF developed in our study was lower than that developed by Attia et al. (13). One possible reason for this performance gap might be the difference in the number of positive AF patients included in the studies (15,419 [8.5%] vs. 2,173 [0.6%]). Fewer AF-positive patients in our dataset might have been caused by ethnic or race differences (30) or lower mean age (48.01 ± 14.72 vs. 60.3 ± 16.7) (31). Another possible reason for the performance gap might be that we used automatic interpretations provided by the ECG machine for defining NSR ECGs. Although it has been shown that the automatic interpretations are most accurate for NSR ECGs, they are still subject to misinterpretations (32). Although a cardiologist reviewed AF ECGs to improve accuracy, reviewing NSR ECGs was not feasible due to the vastness of the dataset. Moreover, considering interobserver variability, having at least two cardiologists review and cross-check the AF ECGs would have provided higher reliability. However, the statistical significance of the regression analysis was obtained even in a less favorable environment (in a model with lower discriminability), and if the model performance had been higher, a more significant result might have been obtained. Third, using AI-ECG-AF, the PAF risk could only be evaluated on NSRs because the AI-ECG-AF was only trained with NSR ECGs. Thus, noncardioembolic IS patients had to be excluded from the study if none of the ECGs measured within seven days before or after the admission date were NSR. To include all ECGs in the analyses, an AI-ECG-AF trained without excluding abnormal ECGs is needed, and we leave this as a subject for further study. Fourth, the patients whose data were included in the regression analyses in our study have not been confirmed whether they really have PAF. This would need prospective validation, which was beyond the scope of our study. However, all the patients were evaluated using the same AI model and the statistical significance achieved is valid at a macro scale. Fifth, the retrospective and single-center design of our study may pose selection bias. The results of our study are yet to be validated in an entirely independent cohort in a prospective manner.

## Conclusion

In conclusion, using the AI-ECG-AF, we found that noncardioembolic IS patients had a higher PAF risk relative to the general patient population. The results from our study imply the need for more vigorous cardiac monitoring in noncardioembolic IS patients. AI-ECG-AF can be a cost-effective screening tool to identify high-risk noncardioembolic IS patients of PAF on-the-spot to be candidates for receiving additional prolonged cardiac monitoring. Our study highlights the potential of AI in clinical practice.

## Data Availability Statement

The original contributions presented in the study are included in the article/Supplementary Material, further inquiries can be directed to the corresponding author/s.

## Ethics Statement

The studies involving human participants were reviewed and approved by Institutional Review Board of Ajou University Medical Center (protocol AJIRB-MED-MDB-21-618). Written informed consent for participation was not required for this study in accordance with the national legislation and the institutional requirements.

## Author Contributions

CH, S-JL, and DY conceived and designed the overall study. CH extracted the ECG data, carried out the statistical analyses, and drafted the manuscript. S-JL and DY made critical revisions to the manuscript. JL, JH, and S-JL generated and extracted the data of noncardioembolic IS patients from the institutional stroke registry. OK preprocessed the data and developed the neural network model. MC reviewed the raw waveforms of all the AF ECGs of patients classified as positive for AF to improve the accuracy of data labeling. OK, MC, SJ, YL, JL, and JH improved the conception and design of the study. All authors revised the manuscript and approved the final version of the manuscript.

## Funding

This work was supported by the Korea Medical Device Development Fund grant funded by the Korean government (the Ministry of Science and ICT; Ministry of Trade, Industry and Energy; Ministry of Health and Welfare; and Ministry of Food and Drug Safety) (Project Number 1711138152 and KMDF_PR_20200901_0095). This study was also supported by a new faculty research seed money grant of Yonsei University College of Medicine for 2021 (2021-32-0044). This work was also partly supported by the Basic Science Research Program through the National Research Foundation of Korea (NRF) funded by the Ministry of Education (NRF-2021R1I1A1A01048331; S-JL).

## Conflict of Interest

OK, MC, SJ, and YL are employees of VUNO Inc. DY is an employee of BUD.on Inc. VUNO Inc. and BUD.on Inc. did not have any role in the study design, analysis, decision to publish, or the preparation of the manuscript. We have no patents, products in development, or marketed products to declare. The remaining authors declare that the research was conducted in the absence of any commercial or financial relationships that could be construed as a potential conflict of interest.

## Publisher's Note

All claims expressed in this article are solely those of the authors and do not necessarily represent those of their affiliated organizations, or those of the publisher, the editors and the reviewers. Any product that may be evaluated in this article, or claim that may be made by its manufacturer, is not guaranteed or endorsed by the publisher.
