# Supplementary material for: Evaluating the Risk of Paroxysmal Atrial Fibrillation in Noncardioembolic Ischemic Stroke Using Artificial Intelligence-Enabled ECG Algorithm
Source: Front Cardiovasc Med. 2022 Apr 8;9:865852. doi: 10.3389/fcvm.2022.865852 (PMC9024295; doi:10.3389/fcvm.2022.865852)
Supplement: Supplementary file 1 [file Data_Sheet_1.DOCX]

Supplementary Material

# Supplementary Methods

## Data preprocessing and AI model development

AUMC’s institutional standard 12-lead ECG database was originally extracted for the June 1994 to May 2020 period from General Electric Healthcare MUSE^TM^ system and stores approximately 1.72 million ECG data. The database consists of the original waveforms measurement data such as heart rate, PR interval, QRS duration, QT interval, personal information such as age, sex, height, and weight, medical departments where the ECG was ordered and automatic ECG interpretations provided by built-in software. The length of each ECG is 10 s and the sampling rate is either 500 Hz or 250 Hz.

Only the raw waveforms of the standard 12-lead ECG were used as the input for the model. ECGs with a sampling rate of 500 Hz were downsampled to 250 Hz, enabling all the ECGs to be arranged in an M $\times$ N matrix, where M is 2500 (the temporal axis; 10 s duration and 250 Hz sampling rate) and N is 12 (the depth-axis; the number of ECG leads). We scaled the data points of each ECG waveform to mean = 0 and standard deviation = 1 (z-score normalization) for stable training.

We constructed a convolutional neural network based on residual networks. Residual networks are composed of skip connections, which allow information to flow directly to the next layer, enabling a much deeper network architecture. Generally, residual networks are believed to have good generalization abilities, and thus, have been used as the backbone architecture in numerous fields. The architecture of our model is depicted in Supplementary Figure 1. Our network was composed of a convolution block, followed sequentially by a max-pooling layer, 17 residual blocks, a global average pooling layer, a fully connected layer, and a softmax layer. The first convolution block was composed of a convolutional layer with a kernel size of seven, a batch normalization layer for the normalization of data distribution, and a rectified linear unit (Relu) activation function for considering the nonlinearity of features. After the input passed the first convolutional block, it passed through a max-pooling layer with a stride 2 and a total of 17 residual blocks. Each residual block comprised two convolutional layers with a kernel size of three. Each of the two convolutional layers was followed by batch normalization and a Relu activation function. The input of each residual block was connected to the output of the second batch normalization layer within the block via skip connection. To reduce sequence length (the temporal axis), a residual block with a stride of two was applied to every two residual blocks from the fourth residual block. The number of feature maps (the depth-axis) doubled every few residual blocks. The network ends with a global average pooling layer, a fully connected layer, and a softmax layer. All the convolutional layers had the “same” padding. AdamW optimizer with an initial learning rate of 0.0001 that used warm-up restarts every 5 epochs were used. An L2 regularization with a coefficient of 0.00001 was applied. The batch size for training was 128. Note that no dropout layers were used. Tensorflow 2.0 was used for implementing the entire architecture and a single graphics processing unit (Titan X Pascal) was used for training. We randomly divided the training dataset into five folds and performed five-fold cross-validation to select the best hyperparameters. As the dataset used in this study was a class imbalanced dataset, in every epoch, we randomly undersampled the majority class in the training dataset to match the sample size of the minority class for stable and balanced training.

## Statistical analyses

P-value < .05 was considered significant in all tests. To check the overall significance or goodness of fit of the regression models, the F-test was conducted in MLiR while the likelihood ratio test was conducted in MLoR. The R-squared values were calculated for MLiR and MLoR to confirm the variance percentage in the dependent variable explained by the independent variables (specifically, Nagelkerke’s R-squared for MLoR). The variance inflation factor (VIF) was calculated for MLiR and MLoR to check for multicollinearity.

In Table 2, statistical significance was obtained for both regression models (F [5, 134123] = 5,983, p < 0.001 for MLiR; chi-square = 15176.2, df = 5, p < 0.001 for MLoR). The variables in MLiR and MLoR explained 18.2% and 15.3% of the overall variance, respectively. Multicollinearity was not present in the data and the VIF for all the variables in both regression models ranged between 1 and 1.1. In Table 3, statistical significance was obtained for both regression models (F [4, 694] = 21.3, p < 0.001 for MLiR; chi-square = 53.4, df = 4, p < 0.001 for MLoR). The variables in MLiR and MLoR explained 11.0% and 9.9% of the overall variance, respectively. Multicollinearity was not present in the data and the VIF for all the variables in both regression models ranged between 1 and 1.2.

Tests for normality of the patient age in each subgroup were conducted (Supplementary Figures 2–3 and Supplementary Table 1). The Shapiro–Wilk test demonstrated that the control group and the “LAA” subgroup could be deemed to have a normal distribution for age, unlike the “cryptogenic” and “SAO” subgroups. The chi-square test comparing the control group, cryptogenic subgroup, LAA subgroup, and SAO subgroup for the confounding variable “sex” was statistically significant (Table 1). The Kruskal–Wallis test comparing the control group, cryptogenic subgroup, LAA subgroup, and SAO subgroup for “age” was statistically significant (Table 1). Post-hoc chi-square tests comparing the control group and each noncardioembolic IS subgroup (i.e., control group vs. cryptogenic subgroup; control group vs. LAA subgroup; control group vs. SAO subgroup) for the confounding variable “sex” with the Bonferroni correction for the 4C2 times of pairwise comparison (i.e., significance level corrected to 0.05/4C2) were statistically significant. Post-hoc independent samples t-tests (two-sided, if the two subgroups being compared could be deemed to have a normal distribution of age) or Mann–Whitney U tests (two-sided, if at least one of the two subgroups being compared did not have a normal distribution of age) comparing “age” between the control group and each noncardioembolic IS subgroup with the Bonferroni correction for the 4C2 times of pairwise comparison were statistically significant. None of the dataset characteristics had statistically significant differences between the training dataset and the control group (the independent samples t-test [two-sided] for the comparison of age; the chi-square test for the comparison of sex or the comparison of NSR ECGs positive for AF).

Supplementary Figure 4 depicts the proportion of males and females by subgroup. Supplementary Figure 5 illustrates the density scatterplot for age and inference output in the control group. The Pearson correlation coefficient was 0.420 and was statistically significant, implying a positive correlation between age and inference output in the control group. Supplementary Figure 6 displays the relative frequency histograms of inference output of each sex in the control group. The Shapiro–Wilk test demonstrated that each sex could be deemed to have a normal distribution for inference output. The independent samples t-test illustrated statistically significant differences of average inference output between each sex (p-value < 0.001). These results imply that age and sex potentially confound the association between patient subgroups and AI-ECG-AF’s inference outputs and need to be adjusted. Differences in the distributions of inference outputs between each subgroup can be observed from the violin plots of inference output for each subgroup (Supplementary Figure 7).

# Supplementary Figures and Tables

## Supplementary Figures

**
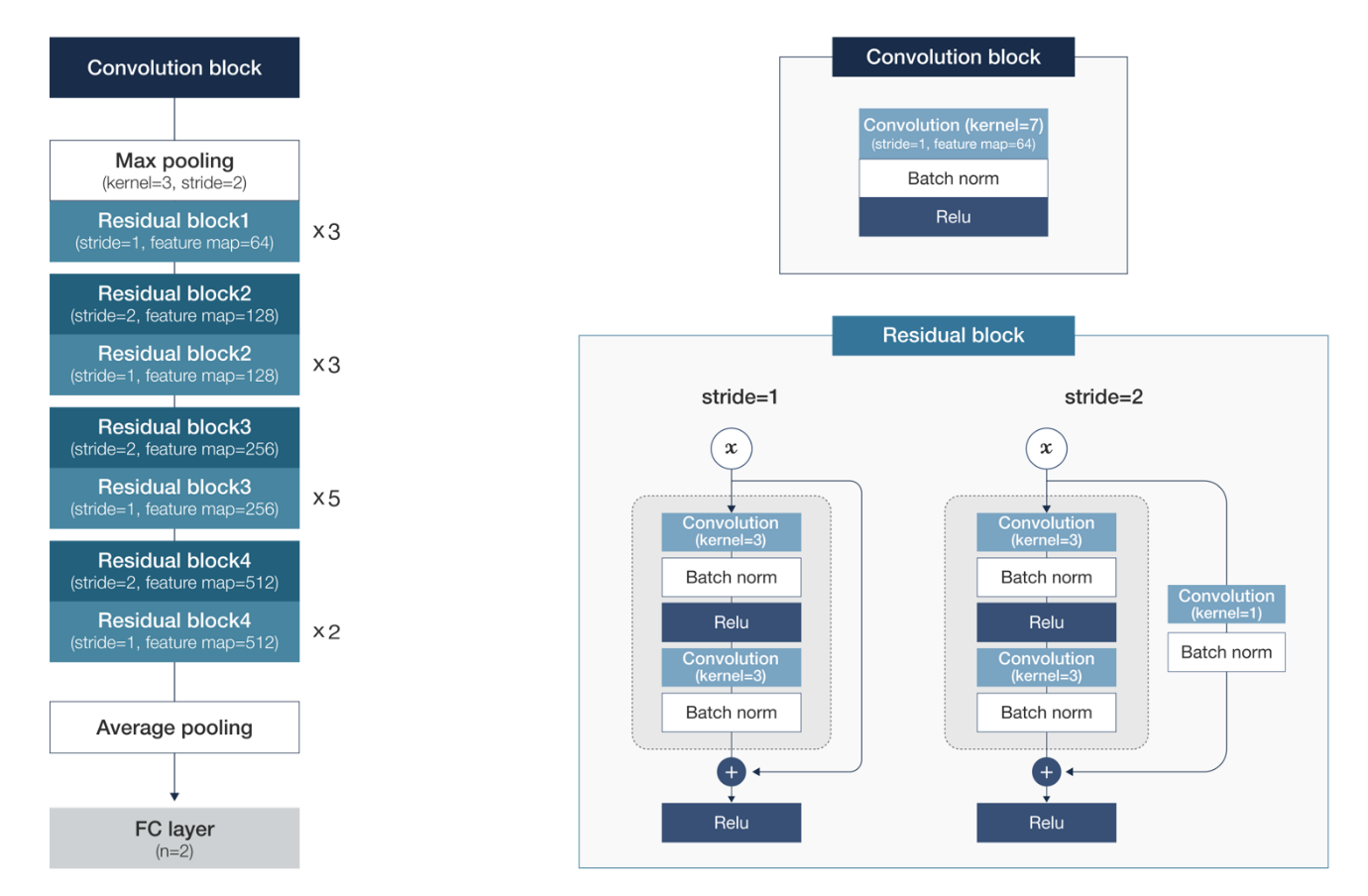
**

**Supplementary Figure 1.** **Architecture of the artificial intelligence model identifying the electrocardiographic signature of atrial fibrillation present during normal sinus rhythm.** FC, fully connected; Relu, rectified linear unit

**
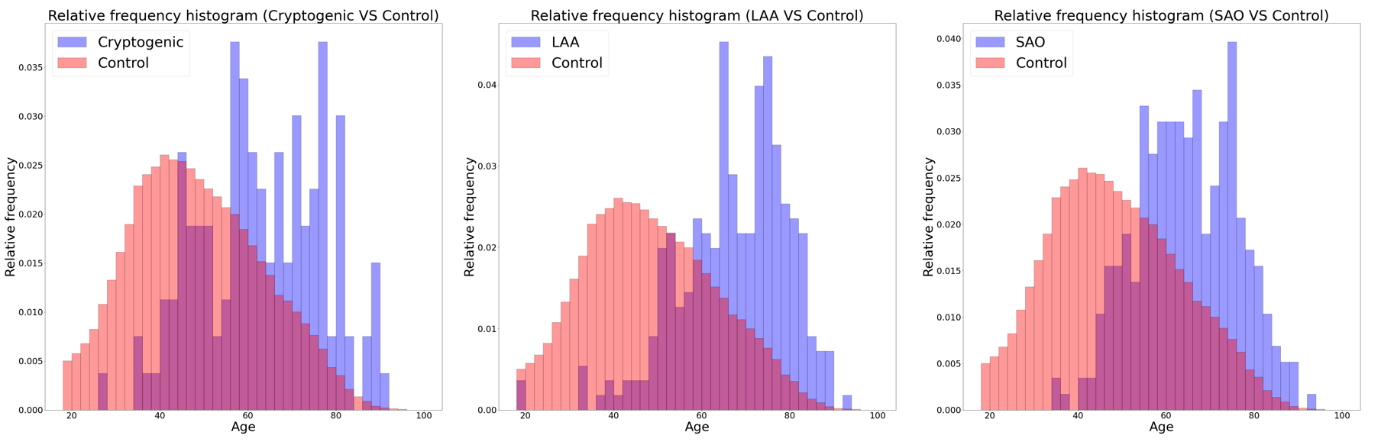
**

**Supplementary Figure 2.** **Relative frequency histograms of age between subgroups.** LAA, large artery atherosclerosis; SAO, small artery occlusion.

**
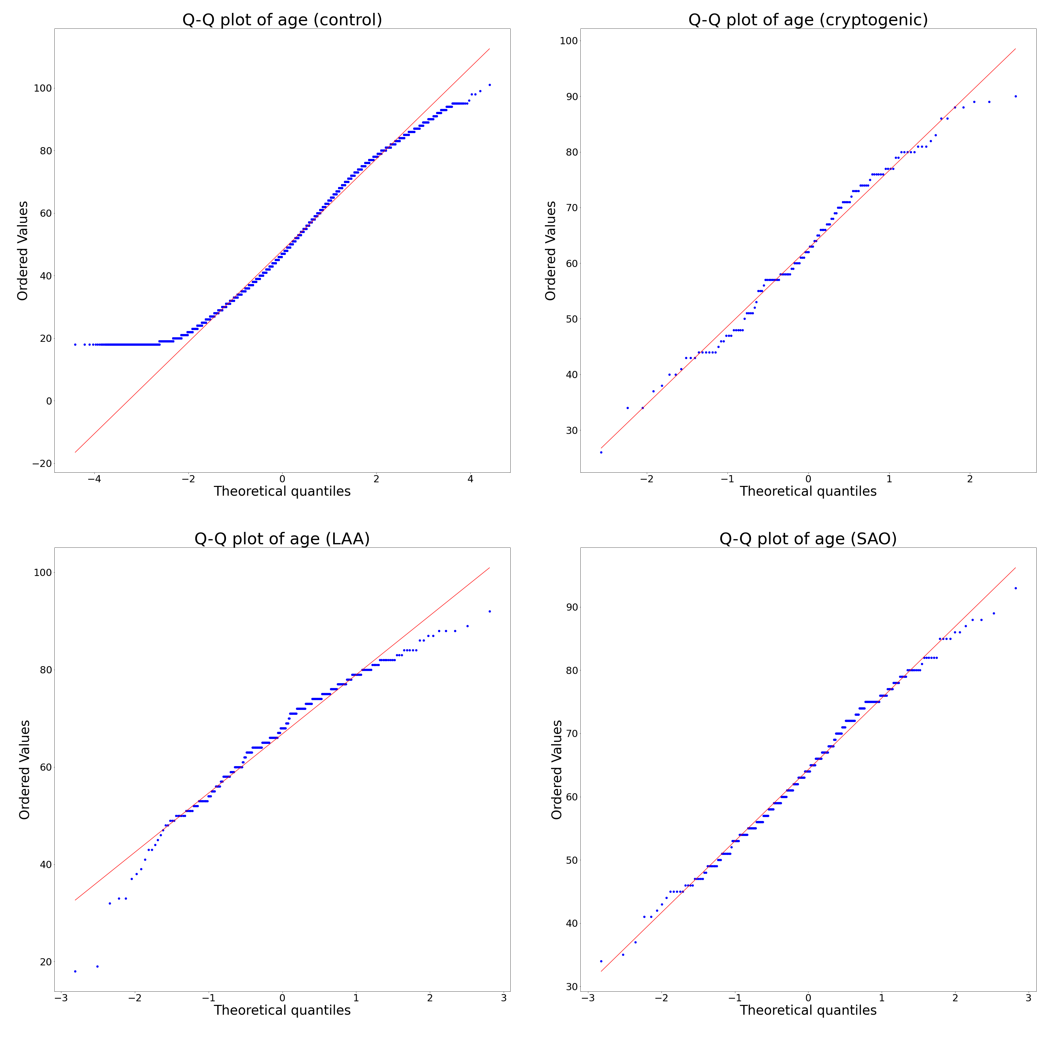
**

**Supplementary Figure 3.** **Q–Q plots of age for each subgroup.** LAA, large artery atherosclerosis; SAO, small artery occlusion.


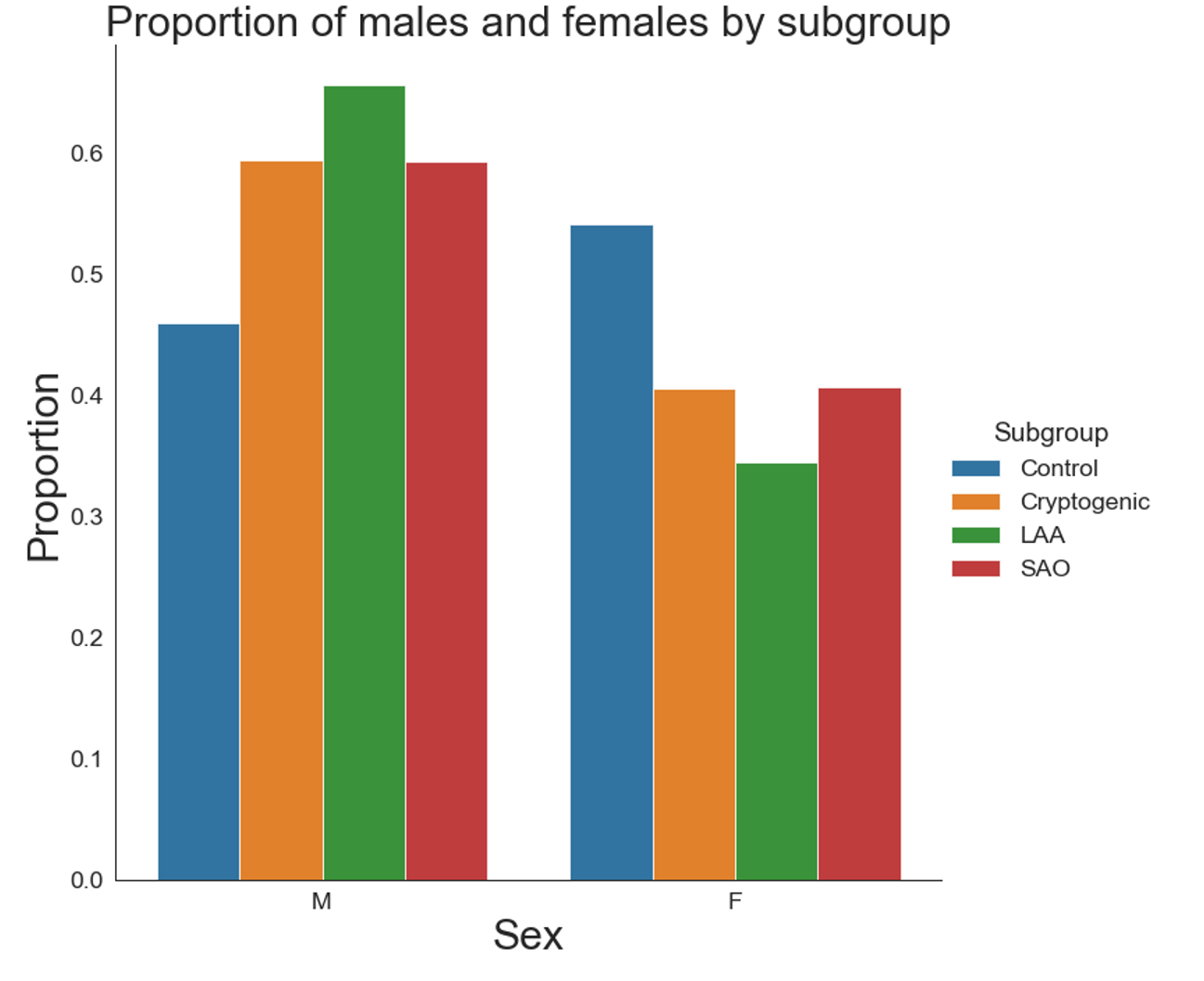


**Supplementary Figure 4.** **Proportion of males and females by subgroup.** LAA, large artery atherosclerosis; SAO, small artery occlusion.


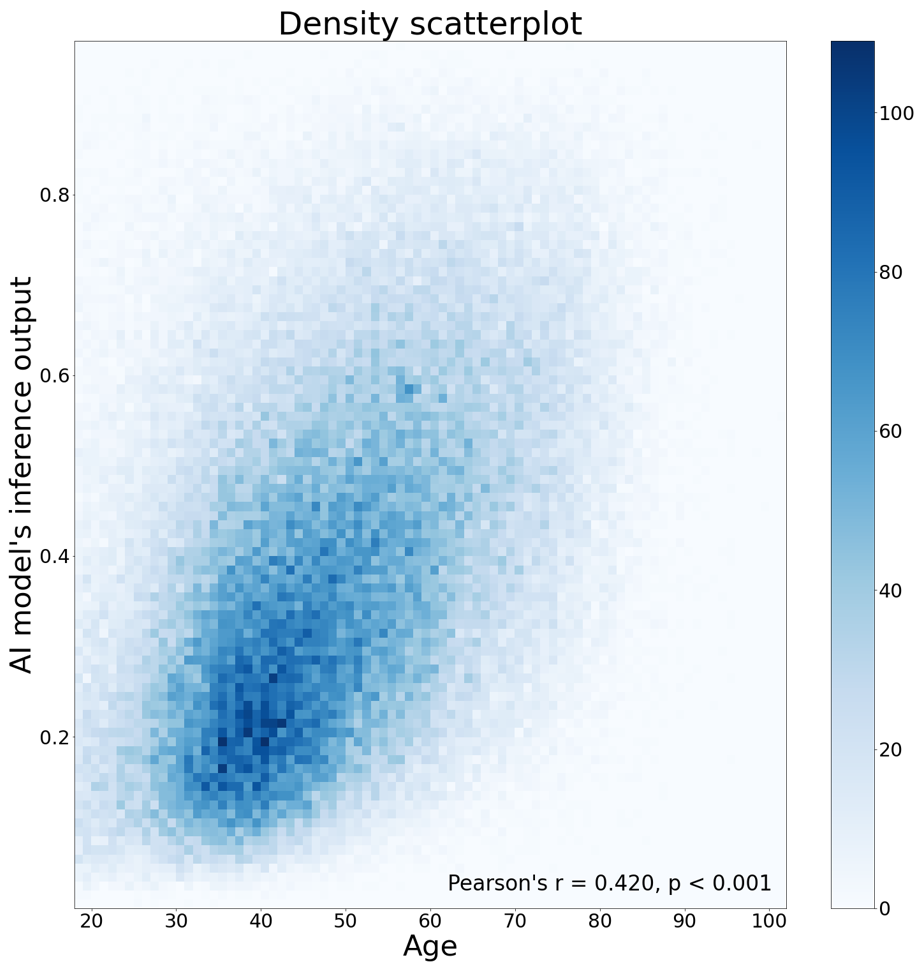


**Supplementary Figure 5.** **Density scatterplot of age and inference output in the control group.** Each pixel represents an age interval of one year and an inference output interval of 0.01. AI, artificial intelligence.


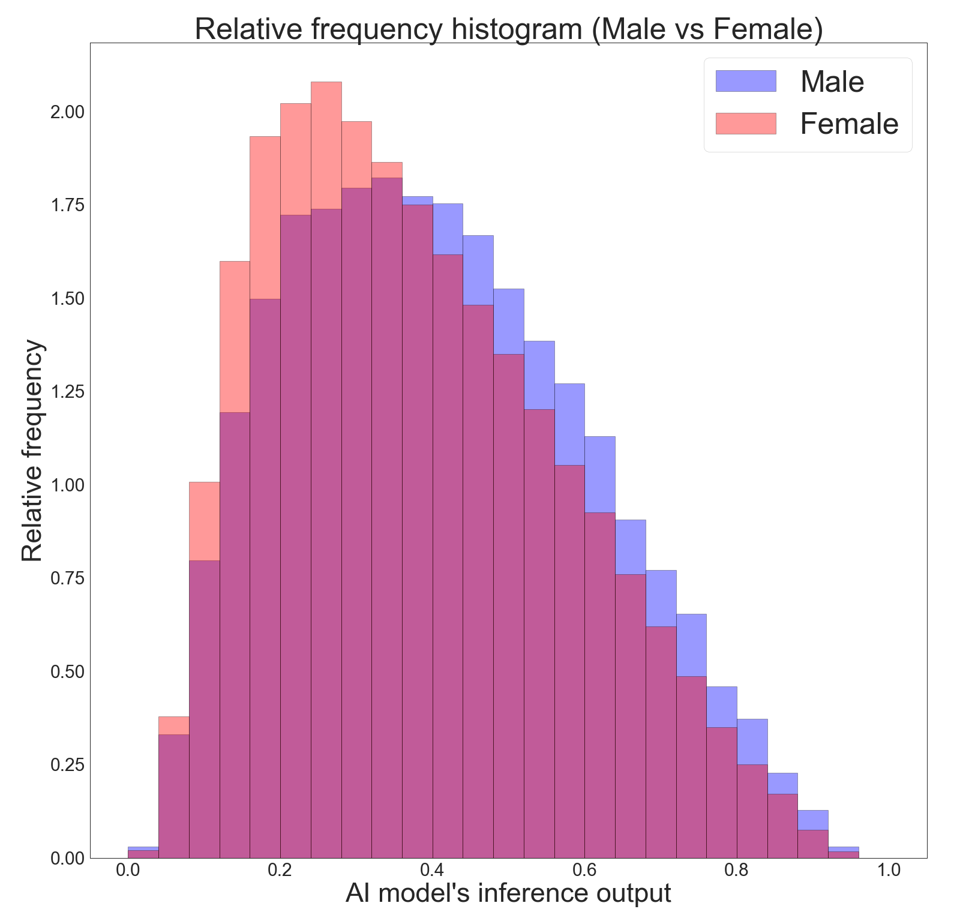


**Supplementary Figure 6.** **Comparison of relative frequency histograms of inference output between each sex in the control group.** The Shapiro–Wilk test demonstrated that each sex could be deemed to have a normal distribution for inference output. The independent samples t-test indicated statistically significant differences of average inference output between each sex (p-value < 0.001). AI, artificial intelligence.


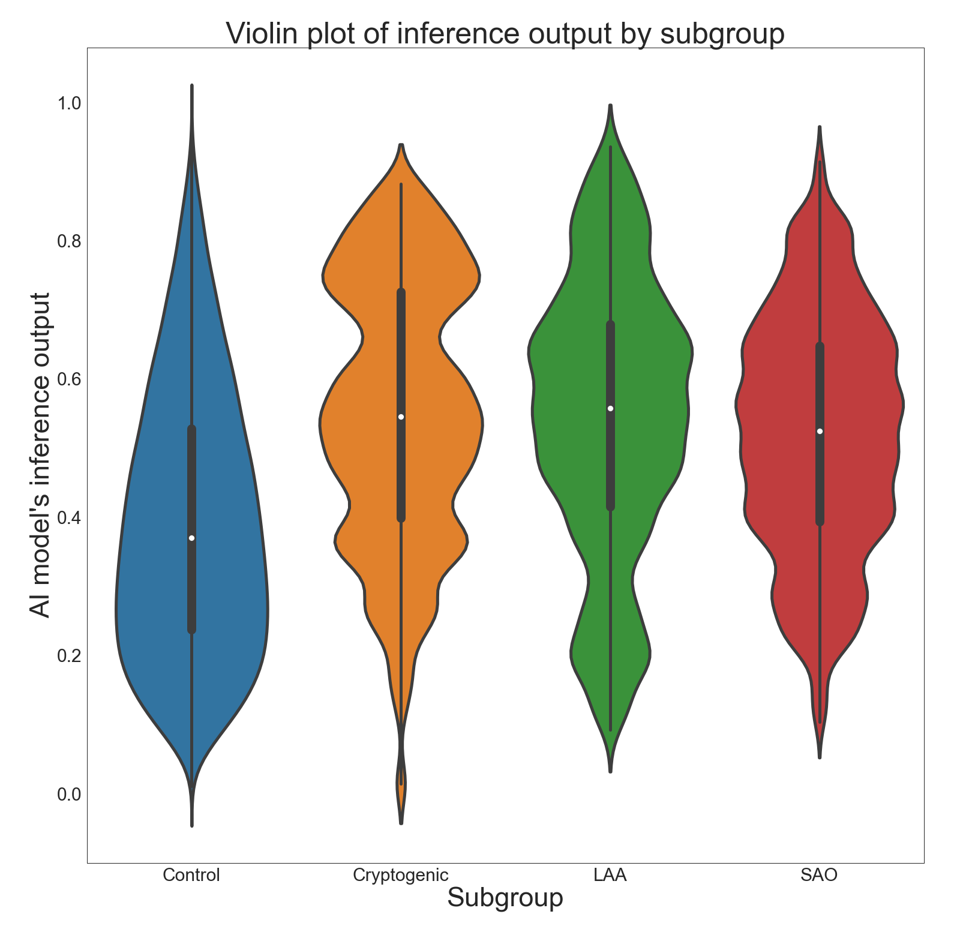


**Supplementary Figure 7.** **Violin plots of inference output for each subgroup.** AI, artificial intelligence; LAA, large artery atherosclerosis; SAO, small artery occlusion.

## Supplementary Tables

**Supplementary Table 1.** **Testing data distributions of age for normality in each subgroup.**

|  | **Skewness** | **Kurtosis** | **p-value (Shapiro–Wilk test)** |
| --- | --- | --- | --- |
| **Control** | 0.272 | −0.523 | < 0.001 |
| **Cryptogenic** | −0.141 | −0.683 | 0.102 |
| **LAA** | −0.780 | 0.970 | < 0.001 |
| **SAO** | −0.061 | −0.513 | 0.175 |

LAA, large artery atherosclerosis; SAO, small artery occlusion.

**Supplementary Table 2.** **Number of ECGs ordered in each medical department in the control group**

|  | **Number of ECGs** |
| --- | --- |
| **Health checkup** | 30,944 (23.2%) |
| **Emergency medicine** | 24,352 (18.3%) |
| **Cardiology** | 10,456 (7.8%) |
| **General surgery** | 6265 (4.7%) |
| **Obstetrics & gynecology** | 5994 (4.5%) |
| **Otolaryngology** | 4716 (3.5%) |
| **Endocrinology** | 4121 (3.1%) |
| **Ophthalmology** | 3003 (2.3%) |
| **Orthopedic surgery** | 2611 (2.0%) |
| **Gastroenterology** | 2603 (2.0%) |
| **Hematology & oncology** | 1905 (1.4%) |
| **Urology** | 1608 (1.2%) |
| **Neurology** | 1410 (1.1%) |
| **Plastic surgery** | 1354 (1.0%) |
| **Family medicine** | 1336 (1.0%) |
| **Other departments** | 6789 (5.1%) |
| **Not specified** | 23,963 (17.8%) |
| **Total** | 133,430 |

ECG, electrocardiogram.

**Supplementary Table 3.** **Performance of the AI-ECG-AF with the threshold set at 0.5.**

|  | **First NSR for each patient in the control group**  **(n = 78,412)** | **All NSRs in the control group**  **(n = 133,430)** |
| --- | --- | --- |
| **Accuracy** | 0.743 | 0.714 |
| **Sensitivity** | 0.674 | 0.654 |
| **Specificity** | 0.743 | 0.715 |
| **PPV** | 0.014 | 0.020 |
| **NPV** | 0.998 | 0.996 |
| **F1 score** | 0.027 | 0.039 |

AI-ECG-AF, artificial intelligence model identifying the electrocardiographic signature of atrial fibrillation present during normal sinus rhythm; NSR, normal sinus rhythm, PPV, positive predictive value; NPV, negative predictive value.

**Supplementary Table 4.** **Multiple logistic regression results at various thresholds for the inference output when the control group and patients with noncardioembolic IS were included in the analyses.** For all the thresholds, the p-value for the likelihood ratio test for overall significance was < 0.001 and multicollinearity was not present in the data and the VIF for all the variables ranged between 1 and 1.1. The variables explained 17.9%, 17.5%, 16.5%, 14.0%, and 12.4% of the overall variance when the threshold for the inference output was set at 0.35, 0.40, 0.45, 0.55, and 0.60, respectively. The inference output for 111 (83.5%), 227 (82.2%), and 236 (81.4%) cases was $\geq$ 0.35 for cryptogenic, LAA, and SAO subgroups, respectively. The inference output for 99 (74.4%), 212 (76.8%), and 217 (74.8%) cases was $\geq$ 0.40 for cryptogenic, LAA, and SAO subgroups, respectively. The inference output for 92 (69.2%), 196 (71.0%), and 186 (64.1%) cases was $\geq$ 0.45 for cryptogenic, LAA, and SAO subgroups, respectively. The inference output for 66 (49.6%), 141 (51.1%), and 133 (45.9%) cases was $\geq$ 0.55 for cryptogenic, LAA, and SAO subgroups, respectively. The inference output for 53 (39.8%), 115 (41.7%), and 98 (33.8%) cases was $\geq$ 0.60 for cryptogenic, LAA, and SAO subgroups, respectively.

| **Threshold** | **Variable** | **OR** | **95% CI of OR** | **p-value** |
| --- | --- | --- | --- | --- |
| 0.35 |  |  |  |  |
|  | Age | 1.056 | 1.056 to 1.058 | < 0.001 |
|  | Sex |  |  |  |
|  | Female | Reference |  |  |
|  | Male | 1.293 | 1.264 to 1.324 | < 0.001 |
|  | Patient subgroup |  |  |  |
|  | Control | Reference |  |  |
|  | Cryptogenic | 2.174 | 1.375 to 3.590 | 0.001 |
|  | LAA | 1.486 | 1.086 to 2.073 | 0.016 |
|  | SAO | 1.599 | 1.189 to 2.188 | 0.003 |
| 0.40 |  |  |  |  |
|  | Age | 1.056 | 1.055 to 1.057 | < 0.001 |
|  | Sex |  |  |  |
|  | Female | Reference |  |  |
|  | Male | 1.298 | 1.268 to 1.328 | < 0.001 |
|  | Patient subgroup |  |  |  |
|  | Control | Reference |  |  |
|  | Cryptogenic | 1.814 | 1.215 to 2.770 | 0.004 |
|  | LAA | 1.585 | 1.086 to 2.073 | 0.002 |
|  | SAO | 1.630 | 1.190 to 2.157 | <0.001 |
| 0.45 |  |  |  |  |
|  | Age | 1.054 | 1.053 to 1.055 | < 0.001 |
|  | Sex |  |  |  |
|  | Female | Reference |  |  |
|  | Male | 1.287 | 1.256 to 1.318 | < 0.001 |
|  | Patient subgroup |  |  |  |
|  | Control | Reference |  |  |
|  | Cryptogenic | 2.046 | 1.397 to 3.045 | < 0.001 |
|  | LAA | 1.731 | 1.324 to 2.285 | < 0.001 |
|  | SAO | 1.432 | 1.118 to 1.842 | 0.005 |
| 0.55 |  |  |  |  |
|  | Age | 1.052 | 1.051 to 1.053 | < 0.001 |
|  | Sex |  |  |  |
|  | Female | Reference |  |  |
|  | Male | 1.281 | 1.247 to 1.316 | < 0.001 |
|  | Patient subgroup |  |  |  |
|  | Control | Reference |  |  |
|  | Cryptogenic | 1.818 | 1.268 to 2.606 | 0.001 |
|  | LAA | 1.535 | 1.199 to 1.965 | 0.001 |
|  | SAO | 1.433 | 1.126 to 1.822 | 0.003 |
| 0.60 |  |  |  |  |
|  | Age | 1.052 | 1.051 to 1.053 | < 0.001 |
|  | Sex |  |  |  |
|  | Female | Reference |  |  |
|  | Male | 1.278 | 1.240 to 1.317 | < 0.001 |
|  | Patient subgroup |  |  |  |
|  | Control | Reference |  |  |
|  | Cryptogenic | 1.781 | 1.231 to 2.560 | 0.002 |
|  | LAA | 1.553 | 1.208 to 1.990 | 0.001 |
|  | SAO | 1.269 | 0.983 to 1.628 | 0.064 |

NSR. normal sinus rhythm; ECG, electrocardiogram; VIF, variance inflation factor; IS, ischemic stroke; SE, standard error, CI, confidence interval; OR, odds ratio; LAA, large artery atherosclerosis; SAO, small artery occlusion.

**Supplementary Table 5.** **Multiple logistic regression results at various thresholds for the inference output when only patients with noncardioembolic IS were included in the analyses.** For all the thresholds, the p-value for the likelihood ratio test for overall significance was < 0.001 and multicollinearity was not present in the data and the VIF for all the variables ranged between 1 and 1.3. The variables explained 12.2%, 13.1%, 12.4%, 9.0%, and 8.0% of the overall variance when the threshold for the inference output was set at 0.35, 0.40, 0.45, 0.55, and 0.60, respectively. The inference output for 111 (83.5%), 227 (82.2%), and 236 (81.4%) cases was $\geq$ 0.35 for cryptogenic, LAA, and SAO subgroups, respectively. The inference output for 99 (74.4%), 212 (76.8%), and 217 (74.8%) cases was $\geq$ 0.40 for cryptogenic, LAA, and SAO subgroups, respectively. The inference output for 92 (69.2%), 196 (71.0%), and 186 (64.1%) cases was $\geq$ 0.45 for cryptogenic, LAA, and SAO subgroups, respectively. The inference output for 66 (49.6%), 141 (51.1%), and 133 (45.9%) cases was $\geq$ 0.55 for cryptogenic, LAA, and SAO subgroups, respectively. The inference output for 53 (39.8%), 115 (41.7%), and 98 (33.8%) cases was $\geq$ 0.60 for cryptogenic, LAA, and SAO subgroups, respectively.

| **Threshold** | **Variable** | **OR** | **95% CI of OR** | **p-value** |
| --- | --- | --- | --- | --- |
| 0.35 |  |  |  |  |
|  | Age | 1.060 | 1.042 to 1.079 | < 0.001 |
|  | Sex |  |  |  |
|  | Female | Reference |  |  |
|  | Male | 1.856 | 1.226 to 2.812 | 0.003 |
|  | IS subgroup |  |  |  |
|  | SAO | Reference |  |  |
|  | Cryptogenic | 1.379 | 0.786 to 2.495 | 0.273 |
|  | LAA | 0.903 | 0.576 to 1.416 | 0.656 |
| 0.40 |  |  |  |  |
|  | Age | 1.061 | 1.045 to 1.079 | < 0.001 |
|  | Sex |  |  |  |
|  | Female | Reference |  |  |
|  | Male | 1.578 | 1.083 to 2.300 | 0.017 |
|  | IS subgroup |  |  |  |
|  | SAO | Reference |  |  |
|  | Cryptogenic | 1.129 | 0.688 to 1.879 | 0.636 |
|  | LAA | 0.948 | 0.631 to 1.424 | 0.796 |
| 0.45 |  |  |  |  |
|  | Age | 1.057 | 1.041 to 1.072 | < 0.001 |
|  | Sex |  |  |  |
|  | Female | Reference |  |  |
|  | Male | 1.297 | 0.916 to 1.836 | 0.143 |
|  | IS subgroup |  |  |  |
|  | SAO | Reference |  |  |
|  | Cryptogenic | 1.439 | 0.908 to 2.308 | 0.125 |
|  | LAA | 1.203 | 0.830 to 1.747 | 0.329 |
| 0.55 |  |  |  |  |
|  | Age | 1.045 | 1.031 to 1.059 | < 0.001 |
|  | Sex |  |  |  |
|  | Female | Reference |  |  |
|  | Male | 1.626 | 1.177 to 2.254 | 0.003 |
|  | IS subgroup |  |  |  |
|  | SAO | Reference |  |  |
|  | Cryptogenic | 1.248 | 0.814 to 1.917 | 0.310 |
|  | LAA | 1.074 | 0.761 to 1.514 | 0.685 |
| 0.60 |  |  |  |  |
|  | Age | 1.043 | 1.029 to 1.058 | < 0.001 |
|  | Sex |  |  |  |
|  | Female | Reference |  |  |
|  | Male | 1.344 | 0.966 to 1.879 | 0.081 |
|  | IS subgroup |  |  |  |
|  | SAO | Reference |  |  |
|  | Cryptogenic | 1.385 | 0.892 to 2.146 | 0.145 |
|  | LAA | 1.244 | 0.874 to 1.770 | 0.225 |

NSR. normal sinus rhythm; ECG, electrocardiogram; VIF, variance inflation factor; IS, ischemic stroke; SE, standard error, CI, confidence interval; OR, odds ratio; LAA, large artery atherosclerosis; SAO, small artery occlusion.
